# Temporal Deletion of AP-2β in Postnatal Mice Demonstrates Distinct Roles in Anterior Angle Development and Intraocular Pressure Homeostasis

**DOI:** 10.1167/iovs.67.12.70

**Published:** 2026-10-28

**Authors:** Lindsey M. Kukoly, Aysha Jamal, Japnit Dham, Serosh Khawaja, Monica Akula, Trevor Williams, Judith A. West-Mays, Aftab Taiyab

**Affiliations:** 1 McMaster University, Health Sciences Centre, Hamilton, Ontario, Canada; 2 Department of Craniofacial Biology, University of Colorado, Aurora, Colorado, United States

**Keywords:** trabecular meshwork, Schlemm's canal, AP-2β, genetics, intraocular pressure, glaucoma

## Abstract

**Purpose:**

The purpose of this study was to investigate how the loss of activating protein 2β (AP-2β) in either the early postnatal or adult mouse eye affects the trabecular meshwork (TM) and intraocular pressure (IOP) homeostasis.

**Methods:**

*UbCre^ERT2^* ^+/−^; *Tfap2b*^−/lox^; *tdTomato*^lox/+^ (UbCre/AP-2β-KO or mutant) and *UbCre^ERT2^*^+/−^; *Tfap2b*^+/lox^; *tdTomato*^lox/+^ (control) pups were exposed to tamoxifen via intraperitoneal injection once daily from postnatal day 4 (P4) to P7 and were euthanized at P14. To investigate adult tissue, tamoxifen exposure occurred from P22 to P25. IOP measurements were taken at P40, P60, or P90 followed by euthanasia. Hematoxylin and eosin (H&E), immunohistochemistry (IHC) and transmission electron microscopy (TEM) were performed to assess the TM, retinal ganglion cells (RGCs), and retinal inflammation.

**Results:**

Loss of AP-2β in early postnatal (P4 to P7) mice showed a partially closed iridocorneal angle and absence of the TM and Schlemm's canal (SC); however, loss of AP-2β in adult mice (P22–P25) produced an open-angle phenotype with preserved outflow pathway structures. Despite maintaining an open-angle phenotype, these mutant mice demonstrated elevated IOP. Ultrastructural analysis revealed flattened vacuoles surrounding the SC, along with pigment dispersion and disrupted extracellular matrix architecture. Additionally, increased IOP was associated with a significant RGC loss, reduced retinal thickness, and increased retinal inflammation.

**Conclusions:**

Early postnatal loss of AP-2β resulted in impaired development of aqueous outflow structures. Loss of AP-2β in adult mice showed an open-angle phenotype, elevated IOP, and marked retinal abnormalities. Together, these outcomes reveal a dual requirement for AP-2β in anterior angle development and TM and IOP homeostasis.

Glaucoma is one of the leading causes of irreversible blindness globally, with 111.8 million individuals projected to be affected by the end of 2040,^1^^,^^2^ and it currently lacks a cure.^1^^–^^3^ The majority of glaucoma cases, including primary angle-closure glaucoma (PACG) and primary open-angle glaucoma (POAG), are associated with increased intraocular pressure (IOP), the pressure generated by aqueous humor in the anterior chamber. Thus, IOP is the major modifiable risk factor for glaucoma. A stable IOP is maintained by a balance between aqueous humor secretion by the ciliary body found behind the iris and aqueous humor outflow through two pathways. The first, the conventional (trabecular) outflow pathway, involves drainage structures in the iridocorneal angle that include the trabecular meshwork (TM) and Schlemm's canal (SC).^4^^,^^5^ The second, the unconventional (uveoscleral) outflow pathway, involves the movement of fluid through the interstitial spaces within the ciliary muscle into the suprachoroidal space, exiting the eye via blood vessels or pores in the sclera leading to the episcleral tissue.^4^^,^^6^^–^^8^ Inhibition of one or both pathways can lead to sustained increases in IOP that exert mechanical stress on the optic nerve (ON) head, ultimately contributing to the progressive loss of retinal ganglion cells (RGCs) and impairment of vision.^9^

Glaucoma can be inherited across generations, indicating that genetics may play a major role in the disease. Multiple genes, such as *PITX2*, *FOXC1*, *FOXC2*, *LTBP2*, and *LMX1B*, have been implicated in glaucoma, including PACG and POAG in humans, and they have been investigated for their role in mouse ocular development and glaucoma.^10^^–^^14^ Another set of genes known to be important for mouse ocular development is the activating protein 2 (AP-2) family of transcription factors, encoded by the *Tfap2* gene family; they are essential for regulating multiple developmental processes and cellular differentiation in the eye.^15^^–^^24^ This family consists of AP-2α (*Tfap2A*), AP-2β (*Tfap2B*), AP-2γ (*Tfap2C*), AP-2δ (*Tfap2D*), and AP-2ε (*Tfap2E*). With specific reference to the iridocorneal angle, AP-2β is expressed in mouse neural crest cell (NCC)-derived periocular mesenchymal (POM) cells beginning around embryonic day 10.5 (E10.5) and has been shown to be required for the formation of aqueous humor drainage structures, including the TM and SC.^22^^,^^23^^,^^25^

AP-2β null mice show ocular defects; however, because these mice do not survive postnatally, they do not allow for assessment of pathology that arises at later stages.^26^ Therefore, we previously used the *Wnt1-Cre* transgenic line to conditionally delete AP-2β from cranial NCCs (AP-2β NCC KOs), thus allowing such mutant mice to be followed into adulthood.^22^ The resulting mice showed anterior segment defects, including the absence of a corneal endothelium and peripheral to central iridocorneal adhesion, as well as an absence in the formation of a TM region (TMR) and SC. These mice also exhibited significantly increased IOP and glaucomatous features.^25^^,^^22^ We have also conditionally deleted AP-2β at a later stage of anterior segment development, after the POM forms, at approximately E14.5, using a *Mgp-Cre* recombinase system. The resultant mutant mice (AP-2β TMR KO) showed an absence of TM and SC, partial angle closure, and impaired retinal function.^23^ These findings illustrate the significance of AP-2β in the development of mouse anterior segment structures including the TM and SC. In corroboration with our studies, a recent analysis using single-cell RNA sequencing from postnatal day 2.5 (P2.5) to P60 mouse anterior segment tissues identified *Tfap2b* as a distinct marker of one out of three TM cell subtypes that are involved in IOP homeostasis.^27^ Furthermore, a recent human genome-wide association study associated *Tfap2b* with POAG, thus indicating a direct link between AP-2β and the incidence of glaucoma in humans.^28^ Taken together, these findings suggest that AP-2β not only may play an important role in development of anterior angle structures such as the TM and SC but may also be required to maintain homeostasis of TM cell function and thus IOP.

In the current study, we investigated whether postnatal deletion of AP-2β, using a tamoxifen-inducible ubiquitin–*Cre* recombinase system (*UbCre^ERT2^*) alters the integrity of the TM region and IOP homeostasis. AP-2β deletion initiated at P4, the stage of POM placement at the anterior angle region,^29^^,^^30^ resulted in impaired development of the TM and SC, with mice exhibiting a partially closed iridocorneal angle, similar to our prior mutant model.^23^ In contrast, conditional deletion of AP-2β at P22, after the anterior angle structures have developed, resulted in an open iridocorneal angle, and the TM and SC were present. However, the ultrastructure of the TM region was altered, and this was accompanied by significantly elevated IOP. Subsequent loss of RGCs and reduced retinal thickness were also observed in these mutants. These findings further demonstrate the involvement of AP-2β in the anterior angle development but, more importantly, also reveal a novel role for AP-2β in regulating TM cell homeostasis and maintenance of IOP in adult mice.

## Materials and Methods

### Animal Husbandry

Animal procedures were completed in accordance with the ARVO Statement for the Use of Animals in Ophthalmic and Vision Research*.* Generation of the tamoxifen-inducible UbCre/AP-2β-KO model was performed using C57BL/6J mice by breeding male mice heterozygous for *UbCre^ERT2^* (*UbCre^ERT2^*^+/−^) with female mice heterozygous for the *Tfap2b* allele (*Tfap2b*^+/−^). From this cross, male mutant mice that possessed the *UbCre^ERT2^* transgene and were heterozygous for the *Tfap2b* allele (*UbCre^ERT2^*^+/−^; *Tfap2b*^+/−^) were generated and subsequently crossed with female mice possessing two floxed *Tfap2b* alleles and the *tdTomato* reporter that serves as a tracer for *Cre* activity (*Tfap2b*^lox/lox^; *tdTomato*^lox/lox^). This cross generated the UbCre/AP-2β-KO mice (*UbCre^ERT2^*^+/−^; *Tfap2b^−^*^/lox^; *tdTomato*^lox/+^) and age-matched control littermates (*UbCre^ERT2^*^+/−^; *Tfap2b*^+/lox^; *tdTomato*^lox/+^) that were used as experimental controls. Genotype was determined using DNA extracted from ear clippings of each mouse, following standard polymerase chain reaction protocols. All animals were housed in groups of five in enriched cages that were cleaned twice a week and kept at 25°C for 12 hours of light and 12 hours of dark.

### Tamoxifen Treatment

UbCre/AP-2β-KO mice and controls were treated with tamoxifen at P4 (pups) or P22 (adults)—that is, before and after the aqueous drainage structures had fully developed. Mice were treated with tamoxifen either through intraperitoneal (IP) injection or as topical eye drops.^31^ The tamoxifen solution was prepared at a concentration of 5 mg/mL for the pups and 10 mg/mL for the adult mice by dissolving tamoxifen powder in corn oil at 37°C, mixing occasionally (Sigma-Aldrich, St. Louis, MO, USA). For the IP injections, P22 to P25 mice were injected with 75 mg tamoxifen/kg of body weight once daily for the treatment period. For the eye drops, tamoxifen was administered topically via eye drops applied three times a day. The P4 to P7 pups received 5-µL eye drops, and 10-µL eye drops were administered to the P22 to P25 adult mice.^31^ UbCre/AP-2β-KO mice and controls were treated with tamoxifen for four consecutive days from either P4 to P7 or P22 to P25. Following this treatment period, tamoxifen exposure was stopped. The P4 to P7 tamoxifen-treated pups were euthanized at P14, and their eyes were enucleated for further analysis. Twelve mutant and 12 control mice were analyzed from the P4 group. All mutant mice expressed the phenotype independent of sex. The P22 to P25 tamoxifen-treated adult mice were monitored and examined until P40, P60, or P90 before euthanasia and enucleation of the eyes for analysis. Sixty mutants and 60 controls from the P22 group were analyzed.

### Intraocular Pressure Measurements

Before taking the IOP measurements, mice were anesthetized through IP injection of 0.06 mL of a ketamine (75 mg) and xylazine (5 mg) mixture per every 10 g of body weight.^32^ Previous investigations have found that ketamine and xylazine can affect IOP readings. To minimize the impact of anesthesia on IOP, the recommended dosage was kept consistent for all mice based on body weight, and readings were taken 10 to 12 minutes following injection to avoid the initial IOP fluctuations.^30^ For the duration of the study, mice were kept on a heating pad set on the low setting to maintain body temperature. IOP measurements were taken between 10:00 AM and 11:00 AM for each group to reduce the risk of circadian effects. Following anesthetic injections, approximately 10 to 12 minutes were allotted for the anesthetic to take effect. IOP readings were obtained using a TonoLab rebound tonometer and aligning the probe to the center of the eye (iCare Finland Oy, Vantaa, Finland).^32^ Readings were taken at P40 (*n* = 34), P60 (*n* = 16), and P90 (*n* = 10). A minimum of six readings were collected for each eye, each of which represented an instrument-derived average value reported by the tonometer. A two-way, repeated analysis of the variance (ANOVA) was conducted to identify changes in IOP overtime using Prism 9.0 (GraphPad Software, Boston, MA, USA).

### Histology

Following euthanasia, eyes were enucleated from KO and control mice. For paraffin sections, eyes were fixed in 4% paraformaldehyde (PFA) for 48 hours. After fixation, a series of three phosphate-buffered saline (PBS) solution washes were conducted, eyes were transferred to 70% ethanol and submitted to the McMaster Histology Department (Core Histology Laboratory, McMaster University, Hamilton, ON, Canada) for processing. Samples were embedded in paraffin wax and sectioned into 4-µm slices. Approximately 90 to 100 sections were obtained for each eye, and those from the center of the eye were selected for hematoxylin and eosin (H&E) and immunofluorescence staining.

For cryosections, eyes were enucleated and fixated in 4% PFA for 1 hour, washed in PBS for 5 minutes, and kept in a 30% sucrose solution overnight. The next day, eyes were transferred to a solution containing 30% sucrose and optimal cutting temperature (OCT) compound for 30 minutes. Next, eyes were embedded in OCT and cryosectioned at 10-µm thickness for immunohistochemical analysis.

### Immunohistochemistry

Sections were deparaffinized in xylene and rehydrated through 100%, 95%, and 70% ethanol, followed by water and treatment with 10-mM sodium citrate puffer (pH 6.0; boiling for 20 minutes) for antigen retrieval. Slides were blocked with 15% normal goat or donkey serum and incubated with primary antibodies (Table) overnight at 4°C. For all colocalization analyses, the two primary antibodies were mixed and incubated simultaneously, followed by a mixture of the two respective secondary antibodies. Slides were mounted with ProLong Gold with 4′,6- diamidino-2-phenylindole (DAPI; Thermo Fisher Scientific, Waltham, MA, USA). All sections were imaged with a Leica DM6 B microscope (Leica Microsystems, Wetzlar, Germany) and observed using LasX 3.4.2.18368 imaging software. Images were reproduced for presentation with image management software. Cell counts were conducted through the Fiji Cell Counter plugin for ImageJ (National Institutes of Health, Bethesda, MD).^33^ Retinal thickness was quantified by Fiji from H&E–stained slides of mice.

**Table.** Antibodies Used for Immunohistochemistry

Antibody	Host	Manufacturer	Dilution
Anti-AP-2β	Rabbit	Cell Signaling Technology	1:50
Anti-ɑ-SMA	Mouse	Sigma-Aldrich	1:100
Anti-Brn3a	Mouse	MilliporeSigma	1:200
Anti-GFAP primary	Mouse	Boehringer Mannheim	1:200
Anti-vimentin	Rabbit	Abcam	1:500
Anti-RBPMS	Rabbit	Abcam	1:2000
Anti-myocilin	Rabbit	FabGennix	1:100
Anti-endomucin	Rat	Abcam	1:100
Alexa Fluor 488 secondary	Goat or donkey	Invitrogen Molecular Probes	1:200
Alexa Fluor 568 secondary	Goat or donkey	Invitrogen Molecular Probes	1:200

### Transmission Electron Microscopy

Enucleated eyes obtained from euthanized P40 mutants and controls were fixed in a solution of 4% PFA and 2% glutaraldehyde for 1 to 2 weeks at 4°C. Samples were sent to the McMaster Electron Microscopy Facility at McMaster University for postfixation in 2% aqueous OsO_4_ and 0.1-M cacodylate buffer at room temperature. Images were visualized through transmission electron microscopy (TEM).

## Results

### Conditional Deletion of AP-2β At an Early Postnatal Stage Causes Partial Anterior Angle-Closure and Decrease in Expression of TM Markers

To understand the requirement of AP-2β in early differentiation of POM into anterior angle structures after the POM cells are placed in the angle region, UbCre/AP-2β-KO mice and controls were topically treated with tamoxifen solution from P4 to P7 to achieve conditional deletion of AP-2β prior to eyelid opening. The eyes were extracted at P14 for histological analysis. Histological evaluation revealed that control P14 mice exhibited an open-angle phenotype and the presence of the TM and SC (Fig. 1). In contrast, mutant mice showed a partial adherence of the iris to the peripheral cornea and an absence of the TM and SC (Fig. 1). The other ocular structures of the anterior segment such as the cornea remained unaltered in the mutant. There was no significant change in corneal thickness in either the P14 group or the P90 group (Supplementary Fig. S1). We further analyzed these tissues by investigating the status of AP-2β expression. In P14 control mice, AP-2β expression was observed in the TM region; however, AP-2β expression was completely absent in the UbCre/AP-2β-KO mice (Fig. 2). Additional immunohistochemistry (IHC) analysis showed the lack of positive staining for alpha smooth muscle actin (α-SMA), a known marker of TM, in the mutant mice, thus providing further evidence of the absence of the TM in these mice (Fig. 3). Myocilin and endomucin staining was used to confirm the absence of the TM and SC in UbCre/AP-2β-KO mice (Supplementary Figs. S2, S3).

**Figure 1.**
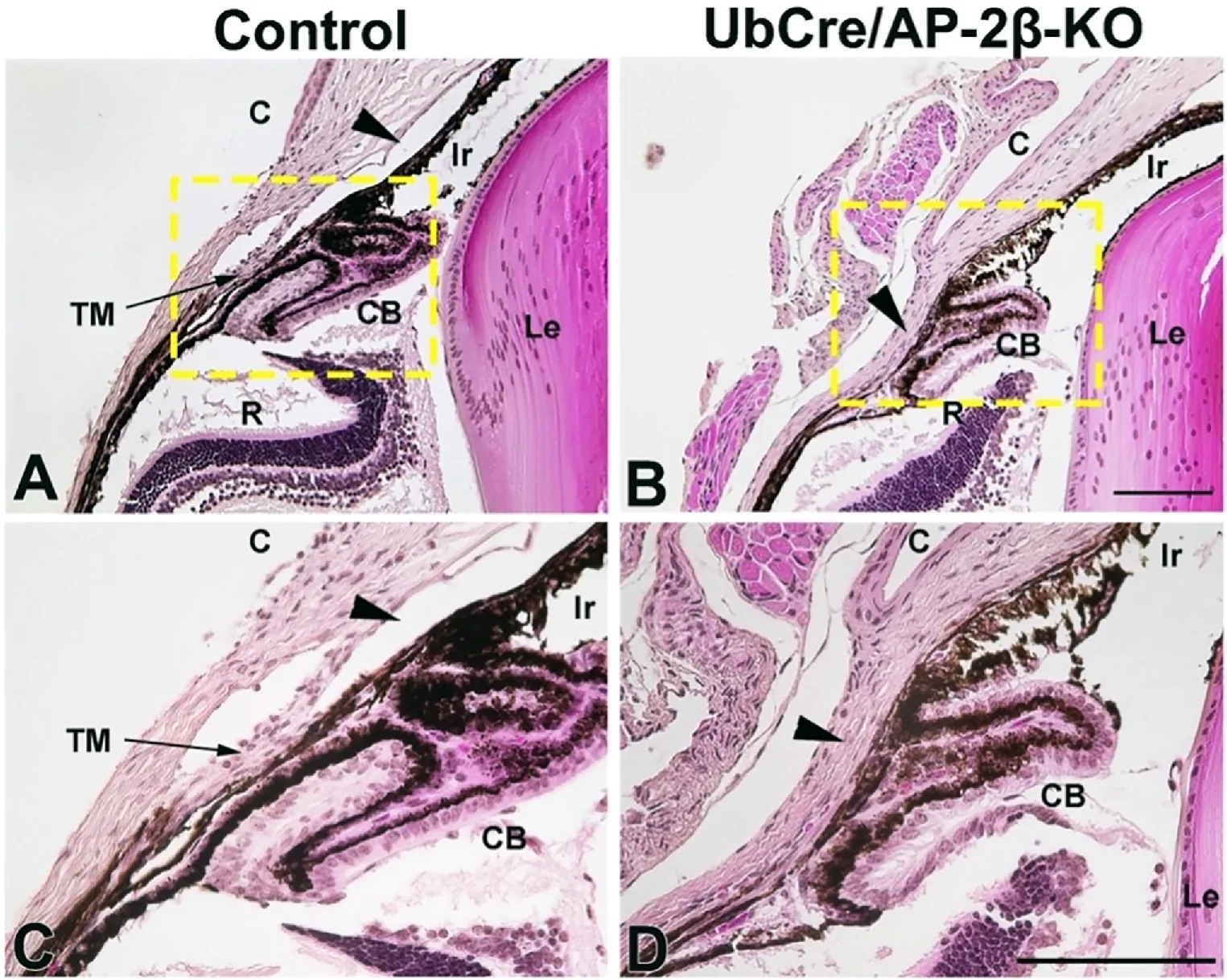
(**A**–**D**) H&E staining of P14 control (**A**, **C**) and UbCre/AP-2β-KO (**B**, **D**) and mice (*n* = 6). P4 to P7 UbCre/AP-2β-KO and controls were topically treated with tamoxifen, and the eyes were enucleated, fixed, and stained with H&E at P14. The *arrowheads* in the control images show the open iridocorneal angle in contrast to the partially closed angle seen in the mutant mice. The *arrows* in the control images show the presence of the TM. In the knockout images, the *arrowheads* show the absence of the TM and SC. The *yellow dotted boxes* show the magnified region. Magnification 20× (**A**, **B**) and 40× (**C**, **D**). *Scale bars*: 100 µm. CB, ciliary body; C, cornea; Ir, iris; R, retina; Le, lens.

**Figure 2.**
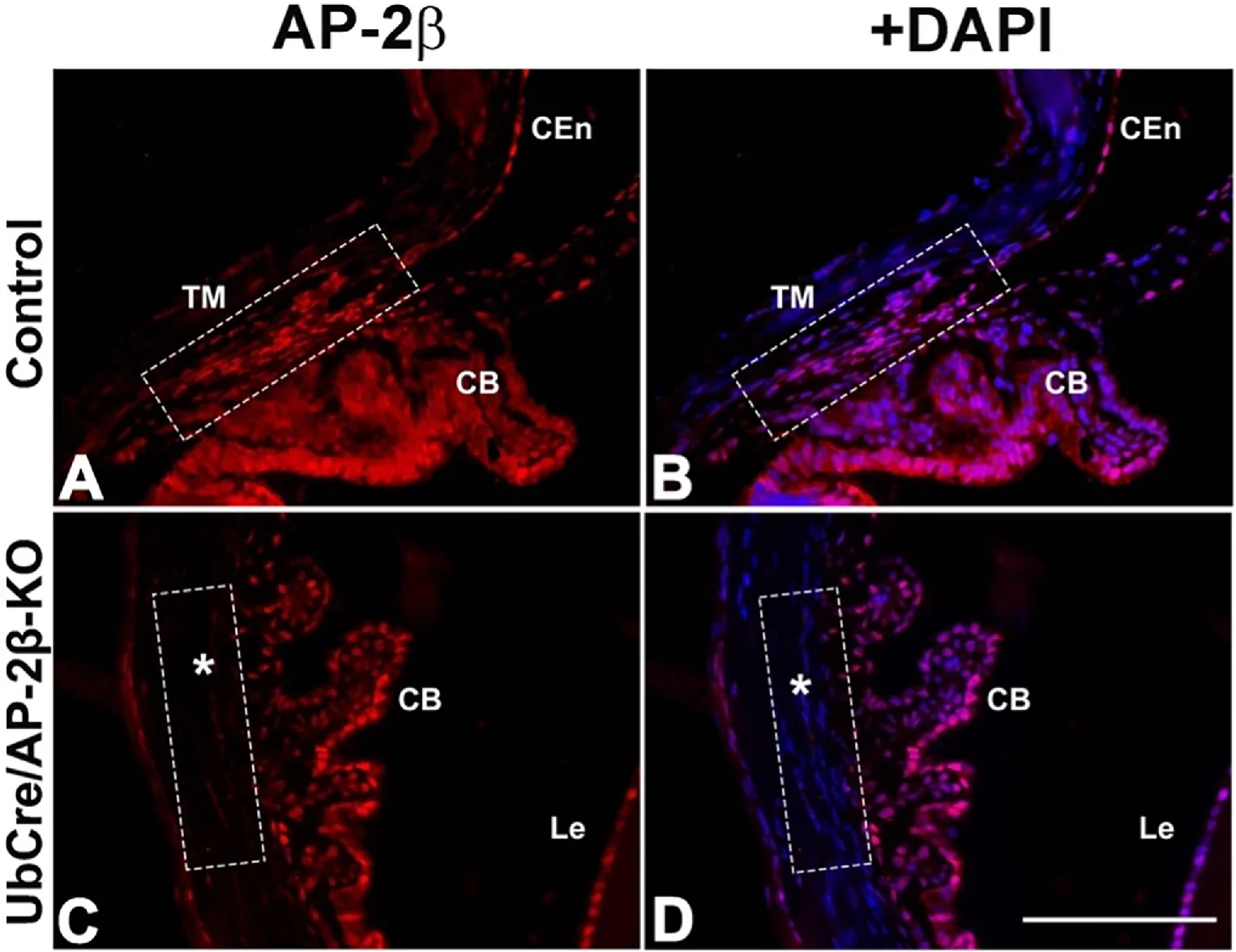
IHC staining for AP-2β (*red*) of UbCre/AP-2β-KO (**C**, **D**) and control (**A**, **B**) mice at P14 (*n* = 3). P4 to P7 UbCre/AP-2β-KO and controls were topically treated with tamoxifen, and the eyes were enucleated, fixed, and stained for AP-2β. The dotted box shows the TM region expressing AP-2β in the control mice. The asterisk marks the absence of AP-2β expression in the TM region of the mutants. Magnification 40×. *Scale bars*: 100 µm. CEn, corneal endothelium; Le, lens.

**Figure 3.**
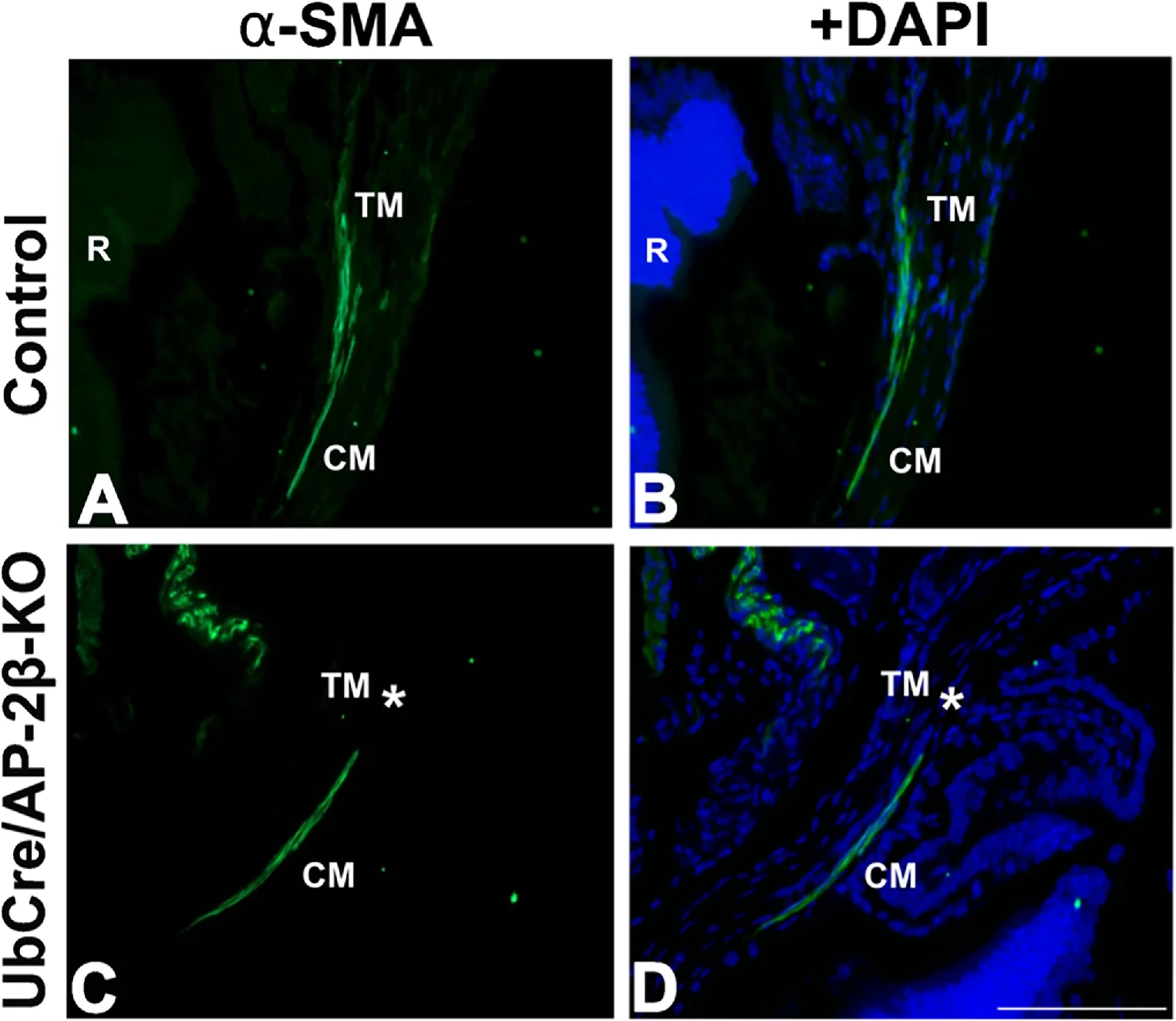
IHC staining for α-SMA-FITC (*green*) in P14 UbCre/AP-2β-KO and control mice (*n* = 3). P4 to P7 UbCre/AP-2β-KO and controls were topically treated with tamoxifen, and IHC for α-SMA was performed. The *asterisks* show the absence of the TM in mutant mice (**C**, **D**) compared to controls (**A**, **B**). Magnification 40×. *Scale bars*: 100 µm. CM, ciliary muscle; CB, ciliary body; R, retina.

### Conditional Deletion of AP-2β in Adult Mice Shows Open Anterior Angle and Reduced Expression of TM Markers

To address the effects of the AP-2β deletion on angle structures and IOP homeostasis in adult mice, UbCre/AP-2β-KO and controls were exposed to tamoxifen solution at P22, the stage following the development of the TM and SC. H&E staining showed an open-angle phenotype and presence of the TM and SC in both the mutant and control mice at P40 (Fig. 4). To determine that tamoxifen treatment is able to facilitate the deletion of floxed allele, we topically treated P22 UbCre/AP-2β-KO-tdTomato and control-tdTomato mice with tamoxifen until P25. The cryosections of P40 UbCre/AP-2β-KO-tdTomato and control-tdTomato revealed no major differences in expression pattern of tdTomato, α-SMA, or myocilin (Fig. 5, Supplementary Fig. S4). Endomucin staining was present in both the control and UbCre/AP-2β-KO mice, thus confirming the presence of the SC (Supplementary Fig. S5). The presence of tdTomato denotes activation of *Cre* transgene in the ocular tissues that were exposed to tamoxifen. However, AP-2β expression was reduced in the TM region of the UbCre/AP-2β-KO mice in comparison to controls at this stage (Fig. 6).

**Figure 4.**
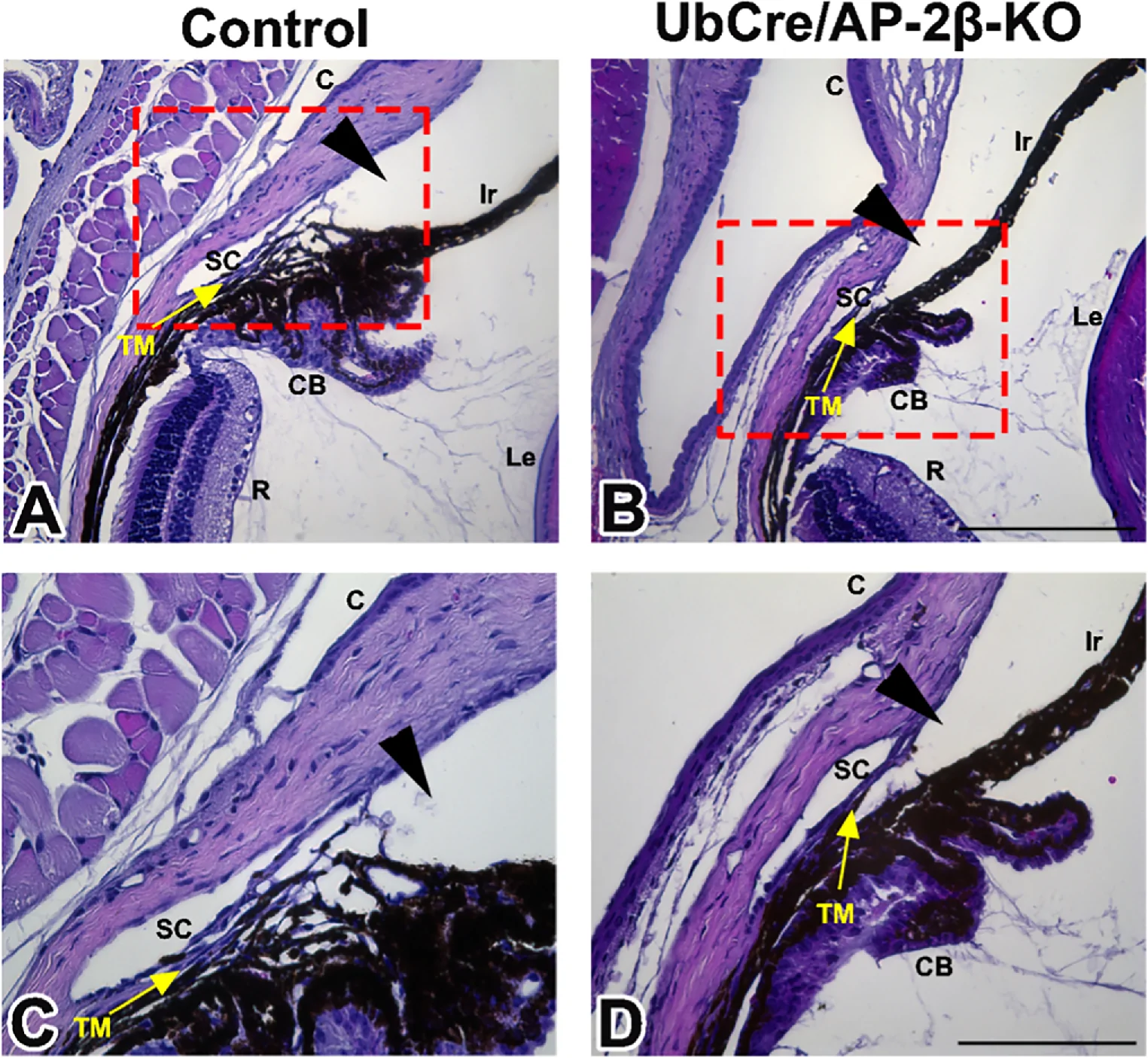
H&E staining of P40 UbCre/AP-2β-KO and control mice (*n* = 6). P22 to P25 UbCre/AP-2β-KO and control mice were topically treated with tamoxifen treatment, and the eyes were enucleated, fixed, sectioned, and stained with H&E. The *arrowheads* show the open iridocorneal angle in both the mutant mice and controls. *Yellow arrows* indicate the TM that is present in both mutant and control mice. The *red dotted boxes* show the magnified region. CB, ciliary body; C, cornea; Ir, iris; R, retina; Le, lens. Magnification 20× (**A**, **B**) and 40× (**C**, **D**). *Scale* *bars*: 200 µm (**A**, **B**) and 100 µm (**C**, **D**).

**Figure 5.**
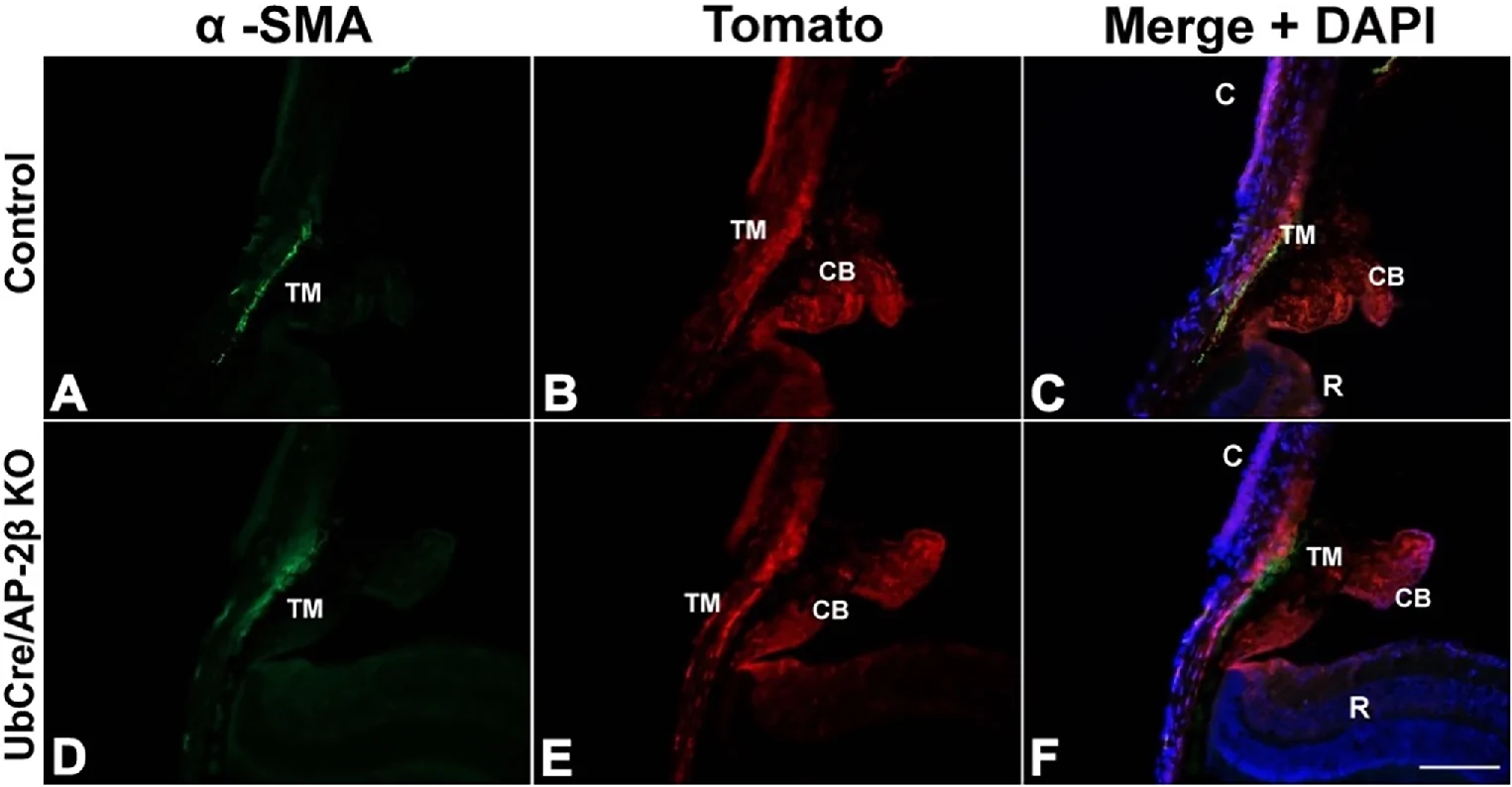
IHC staining for α-SMA-FITC (*green*) and imaging for α-SMA and tdTomato (*red*) in P40 UbCre/AP-2β-KO (**D**–**F**) and control mice (**A**–**C**) (*n* = 3). P22 to P25 UbCre/AP-2β-KO and control mice were topically treated with tamoxifen, and the eyes were enucleated, fixed, sectioned, and stained for α-SMA. Staining shows the presence of the TM in both the control and mutant mice. Magnification 40×. *Scale bars*: 100 µm.

**Figure 6.**
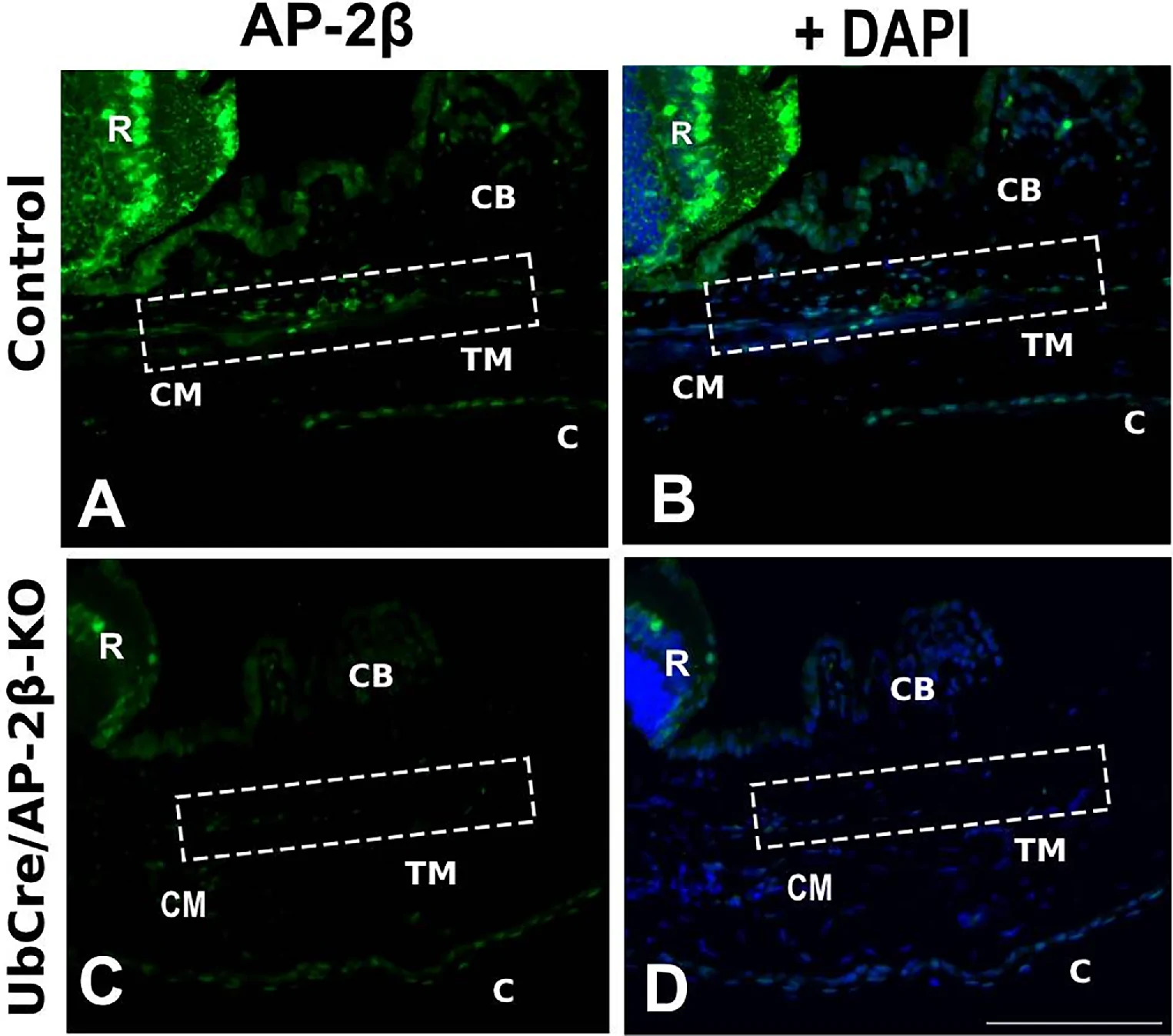
AP-2β staining of UbCre/AP-2β-KO (**C**, **D**) and control (**A**, **B**) mice at P40 (*n* = 3). P22 to P25 UbCre/AP-2β-KO and control mice were topically treated with tamoxifen, and the eyes were enucleated, fixed, sectioned, and stained for AP-2β. Staining shows AP-2β expression in the TM region of both control and mutant mice, denoted by the *asterisks*. Magnification 40×. *Scale bars*: 100 µm.

### UbCre/AP-2β-KOs Show Increased IOP

To examine whether deletion of AP-2β in the TM region of UbCre/AP-2β-KO adult mice resulted in changes in IOP we performed rebound tonometry on the mice. Figure 7 shows the average IOP of control and mutant mice at P40, P60, and P90. At each age, the mutant IOP was significantly higher compared to the control values (*P* < 0.0001). However, no significant changes in IOP were noted within the mutant mice between each postnatal stage examined.

**Figure 7.**
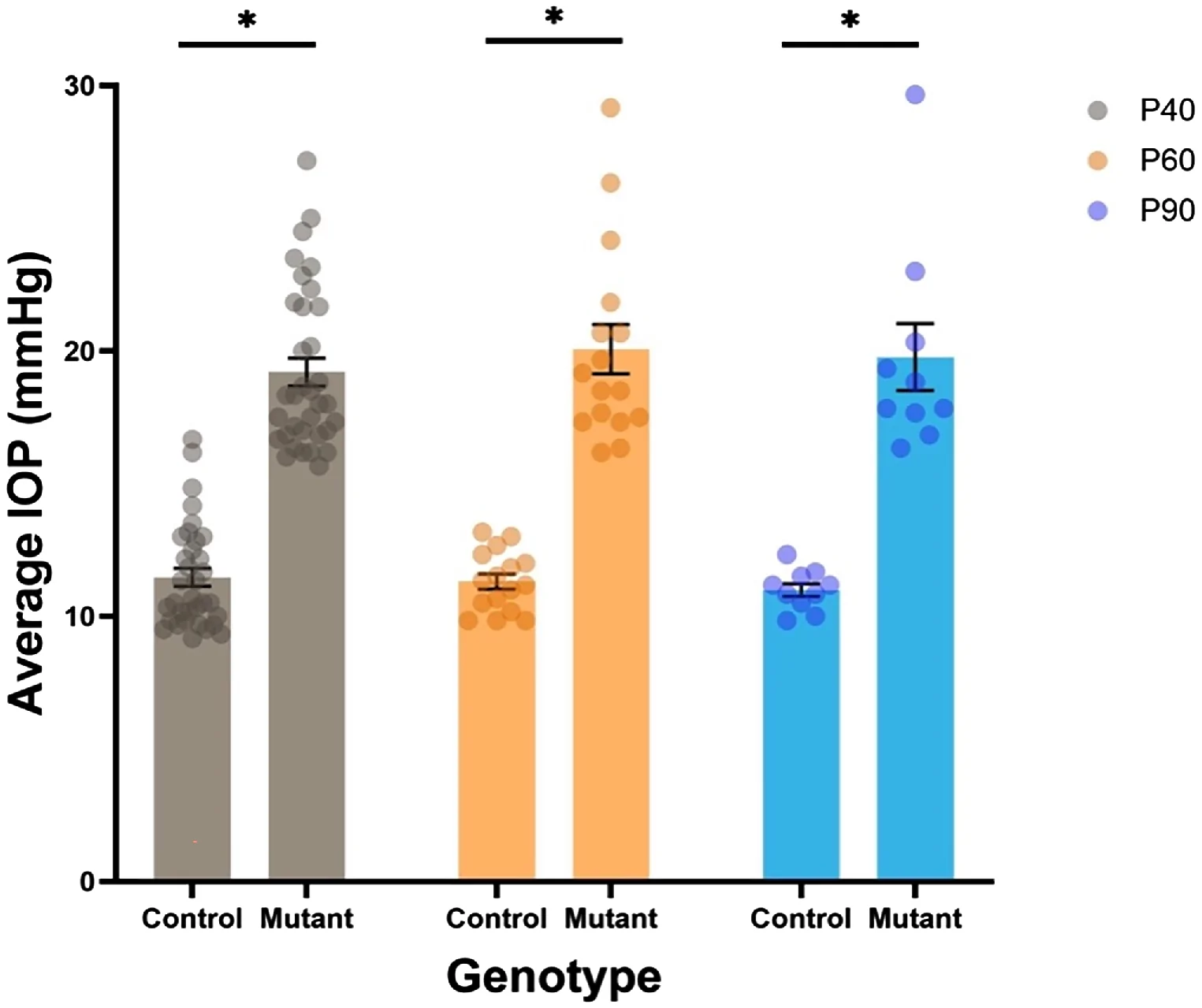
Average IOP of UbCre/AP-2β-KO mice compared to controls. The animals were injected with tamoxifen P22 to P25, and the IOP was measured at P40, P60, and P90. The *gray* *bars* show the average IOP for each group at P40 (*n* = 34 eyes). The *orange bars* show the average IOP of each group at P60 (*n* = 16 eyes), and the *blue bars* show the average IOP at P90 (*n* = 10 eyes). One eye from each mouse was included in these groups. *Error bars* represent the standard error of the mean (SEM), and the *asterisks* represent *P* values (*P* < 0.0001 for each time point).

### AP-2β Expression Levels Differentially Regulate IOP

The relative deletion of AP-2β expression was evaluated in relation to IOP. A marked reduction in AP-2β expression was observed in UbCre/AP-2β-KO mice compared with their control littermates (Fig. 8). However, expression levels among mutant mice demonstrated some variability that interestingly corresponded to differences in IOP. For example, mutant mice with higher IOP readings exhibited a greater reduction in AP-2β expression, whereas lower IOP values were associated with higher AP-2β expression within the TM region (Fig. 8). To further quantify the relationship between the AP-2β expression and IOP, mutant mice were categorized into low-IOP and high-IOP groups, with high IOP defined as values ≥16 mm Hg (Supplementary Fig. S6). At P60, UbCre/AP-2β-KO mice demonstrated significantly reduced AP-2β expression within the TM region compared with controls (Supplementary Fig. S6). This reduction was observed in both low- and high-IOP mutants, with a greater decrease in AP-2β expression in the high-IOP group (Supplementary Fig. S6). These findings suggest that the modulation of AP-2β expression within the TM region strongly correlates with changes in IOP levels.

**Figure 8.**
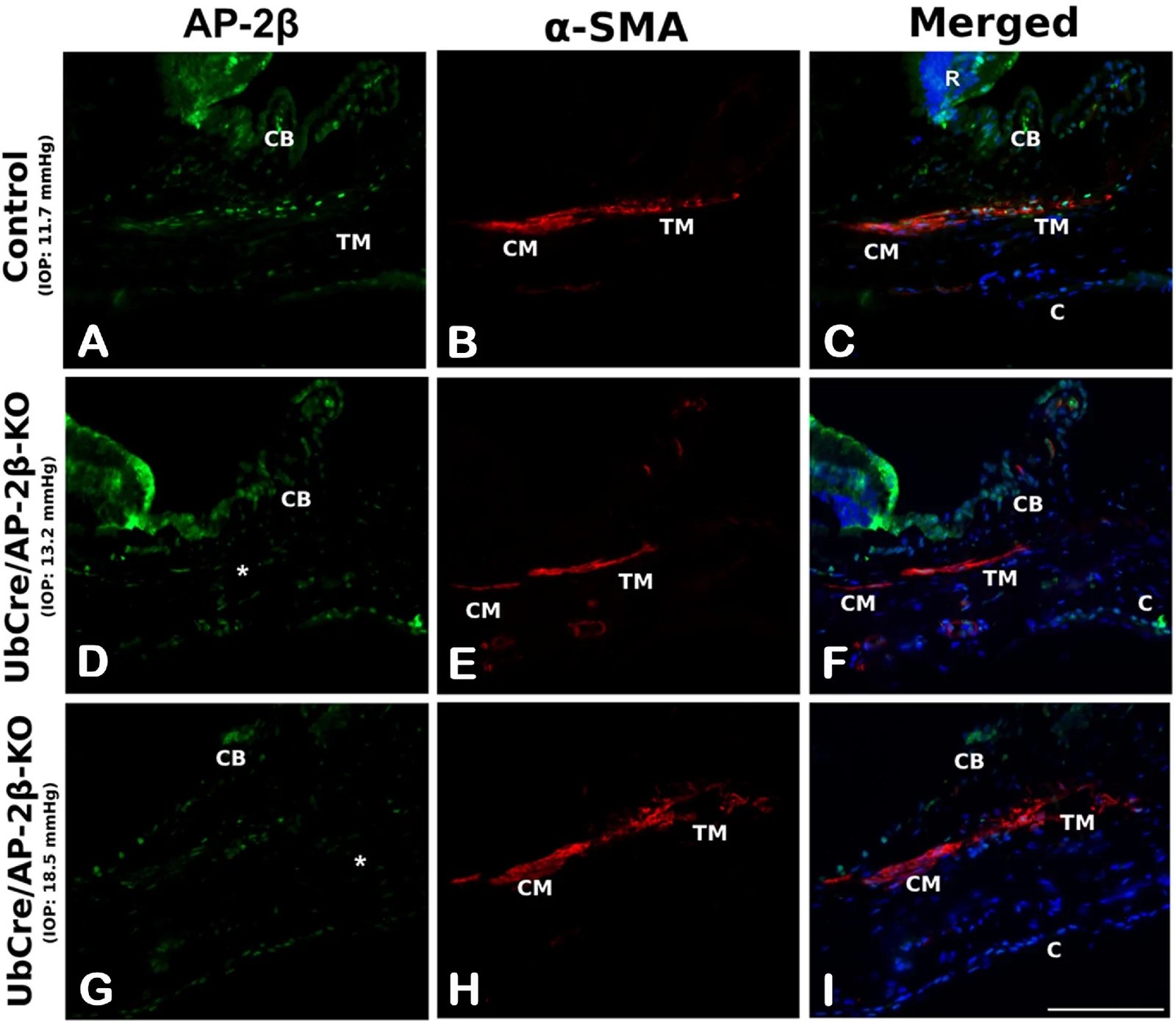
AP-2β (*green*) and α-SMA (*red*) staining in the anterior angle of control and UbCre/AP-2β-KO mice at P60. P22-P25 UbCre/AP-2β-KO and control mice were injected with tamoxifen and the eyes were enucleated, fixed, sectioned and stained for AP-2β. (**A**–**C**) Control mice (IOP:11.7 mm Hg) show strong AP-2β expression within the ciliary body and TM, whereas UbCre/AP-2β-KO mice (IOP:13.2 mm Hg (**D**–**E**) and 18.5 mm Hg (**G**–**I**)) exhibit reduced AP-2β expressed within the targeted region. *n* = 3. The *asterisk* (*) marks AP-2β expression in the TM region. Magnification 40×. *Scale bar*: 100 µm.

### Loss of AP-2β Leads to Changes in Anterior Angle Structure

To perform a detailed evaluation of structural changes within the anterior segment, we performed TEM on P40 UbCre/AP-2β-KO mutant and control eyes. In P40 UbCre/AP-2β-KO mice, the anterior segment retained an open iridocorneal angle similar to that of control littermates (Figs. 9A, 9E). We noted that the pigment arrangement differed between the control and mutant mice, where pigments appeared clustered in controls and more dispersed in the mutant (indicated by the asterisks in Figs. 9B, 9F). At a higher magnification, mutant eyes exhibited flattened vacuoles surrounding SC in contrast to the control mice where giant vacuoles were present and distended (indicated by arrows in Figs. 9C, 9G). Additionally, TM cells were sparser in mutant mice compared to controls, collagen fibers appeared fragmented, and the extracellular matrix (ECM) components of the TM region in mutant eyes appeared more disorganized and irregular (arrowheads in Figs. 9D, 9H).

**Figure 9.**
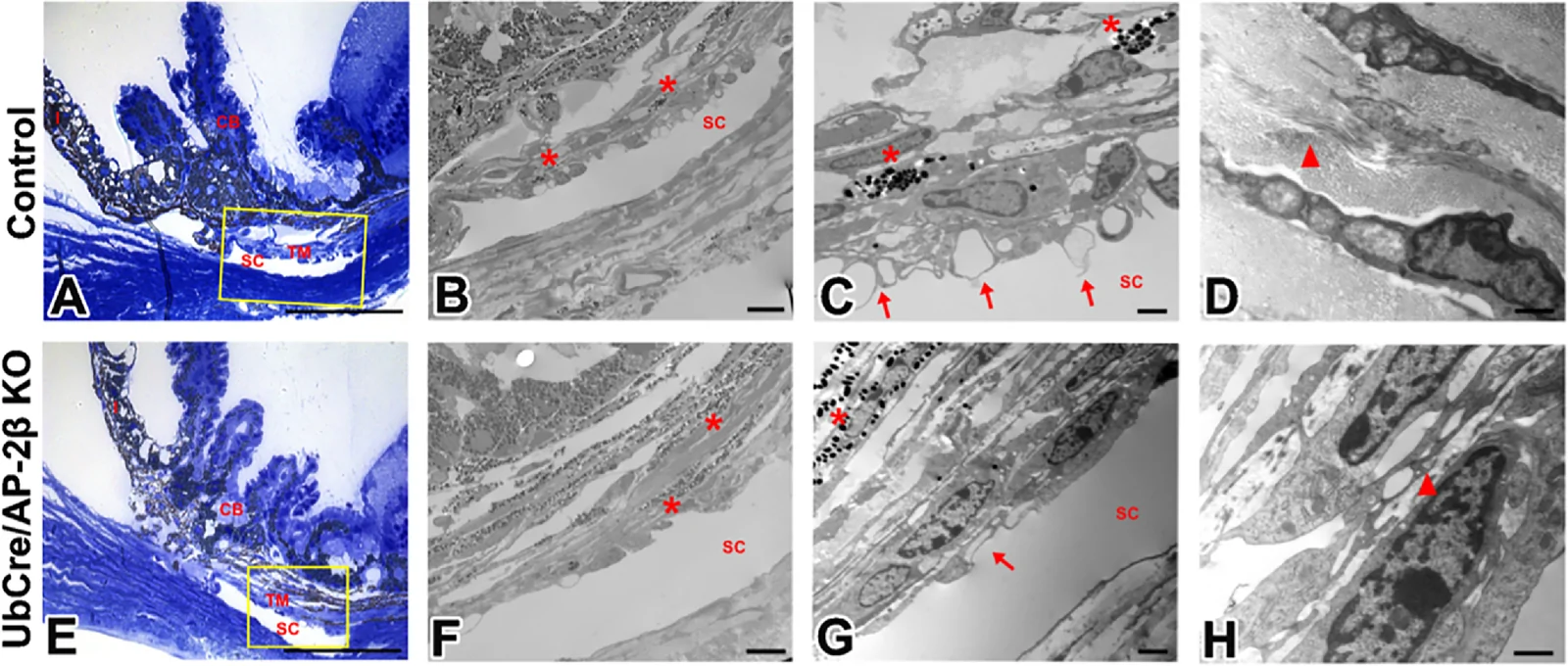
(**A**, **E**) Toluidine blue staining shows open iridocorneal angles in P40 control and UbCre/AP-2β-KO mice. Magnification 40×. *Scale bar*: 100 µm. P22 to P25 UbCre/AP-2β-KO and control mice were injected with tamoxifen, and the eyes were enucleated, fixed, and processed for TEM. The *yellow boxes* show the magnified region. TEM images highlight the ultrastructure of the TM. (**B**) At 1500× magnification, the *red asterisks* mark pigments organized into clusters in the controls. *Scale bar*: 10 µm. (**F**) In the mutants, pigments are dispersed. Magnification 2000×. *Scale bar*: 8 µm. (**C**, **G**) At 6000× magnification, the *arrows* indicate normal distended giant vacuoles in control eyes compared with flattened vacuoles in UbCre/AP-2β-KO mice. *Scale bar*: 2 µm. (**D**, **H**) At 20,000× magnification, the *arrowheads* mark ECM components, which appear more intact in control tissue and more disorganized in the mutants. *Scale bar*: 800 nm. Control, *n* = 3; mutant, *n* = 3.

### Elevated IOP Correlates With Glaucomatous Changes in UbCre/AP-2β-KO Eyes

An increase in IOP is known to induce changes in the retina such as RGC death, a hallmark of glaucoma progession.^9^ Therefore, to determine whether increased IOP in UbCre/AP-2β-KO mice was associated with additional glaucomatous changes, such as loss of RGCs we used RNA binding protein with multiple splicing (RBPMS), a marker of RGCs, to quantify RGC numbers and compared that with total number of cells present in the ganglion cell layer (GCL). Total cell counts were derived from all DAPI-positive nuclei in the GCL, representing both RGCs and non-RGC cell types. Because RGC loss is a known downstream consequence of elevated IOP,^34^ we examined RGC numbers in retinal sections from P60 and P90 control and UbCre/AP-2β-KO mice. UbCre/AP-2β-KO retinal sections from both stages exhibited significant, overall reduction in RBPMS-positive cells compared with controls (Figs. 10, 11). At P60, the total number of cells in the GCL did not differ between controls and UbCre/AP-2β-KO mice, showing mean counts of 208 ± 11 cells and 224. ± 17 cells, respectively (*P* = 0.3410) (Fig. 10B). In contrast, a significant reduction in RBPMS-expressing cells was observed in the mutants, where controls exhibited 151 ± 8 RBPMS⁺ cells compared to 109 ± 4 in UbCre/AP-2β-KO, thus showing a significant decrease in RBPMS-expressing cells in UbCre/AP-2β-KO mice (*P* < 0.05) despite preserved overall cell number in the GCL (Fig. 10B). When assessing the proportion of RBPMS⁺ cells relative to the total GCL population, controls displayed 70.35% ± 0.98% in the peripheral retina, 74.84% ± 0.90% centrally, and 72.54% ± 0.49% overall, whereas the proportion was significantly lower in mutants, reported as 48.91% ± 1.94% (*P* < 0.0001), 52.34% ± 2.33% (*P* < 0.0001), and 48.92% ± 3.46% (*P* < 0.0001), respectively (Fig. 10C). These findings demonstrate that, although total GCL cell numbers remain unchanged at P60, the percentage of RBPMS-expressing RGCs is significantly reduced in UbCre/AP-2β-KO mice (Fig. 10).

**Figure 10.**
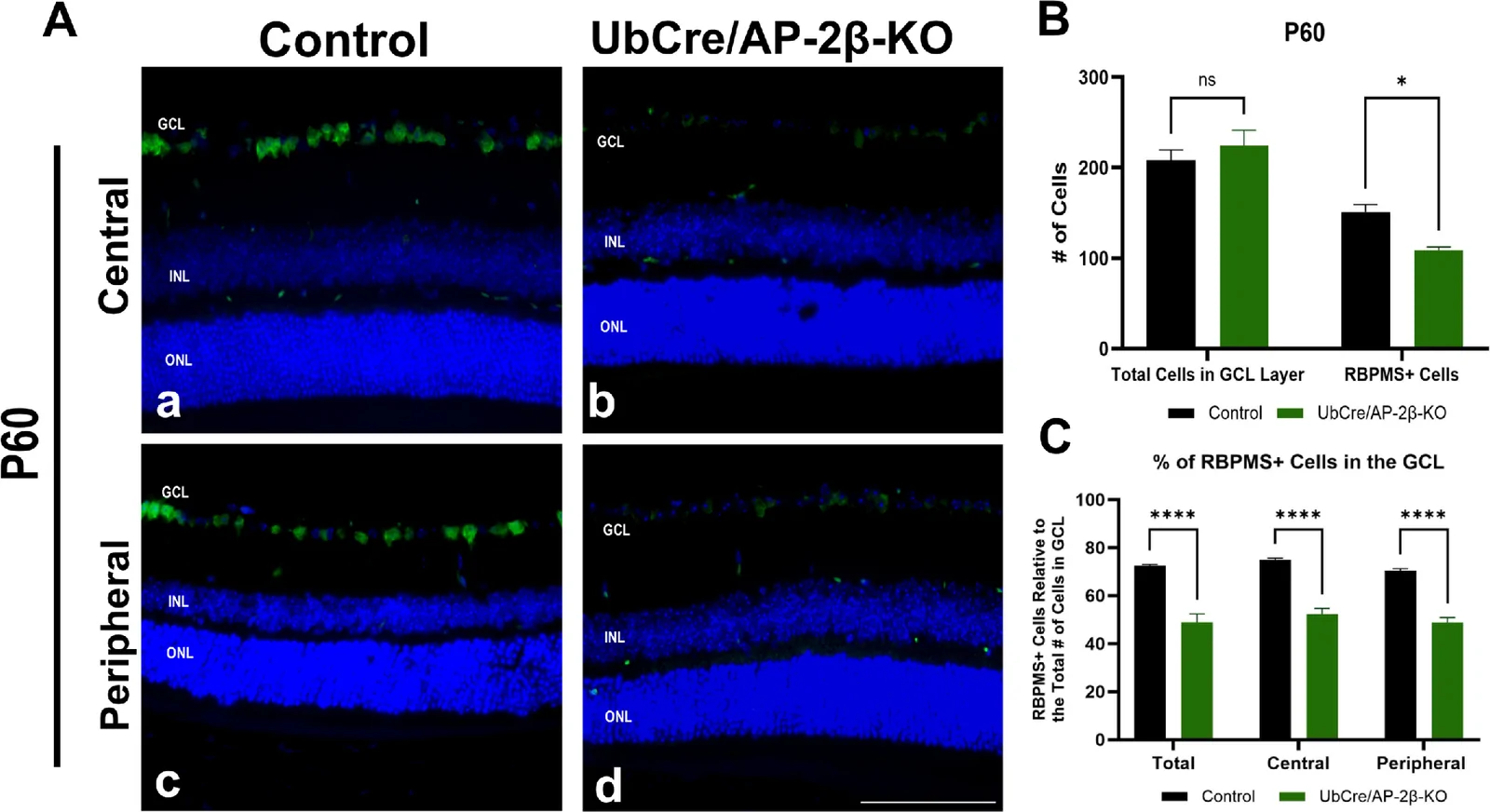
RBPMS and cell counts at P60 (*n* = 3). P22 to P25 UbCre/AP-2β-KO and control mice were injected with tamoxifen, and the eyes were enucleated at P60, processed, sectioned, and stained for RBPMS. (**A**) Immunofluorescence images of central and peripheral retinal sections from control and UbCre/AP-2β-KO mice at P60 stained for RBPMS (*green*) and nuclei (*blue*). (**B**, **C**) Quantification shows a reduction in RBPMS⁺ RGCs in UbCre/AP-2β-KO mice. Bar graphs display the total number of cells in the GCL layer (**B**) and the number and proportion of RBPMS⁺ cells (**C**) for P60. A two-way ANOVA was performed, and data were graphed using GraphPad Prism. *Error bars* represent SEM. *P < 0.05, ****P < 0.0001. Magnification 40×. *Scale bar*: 100 µm.

**Figure 11.**
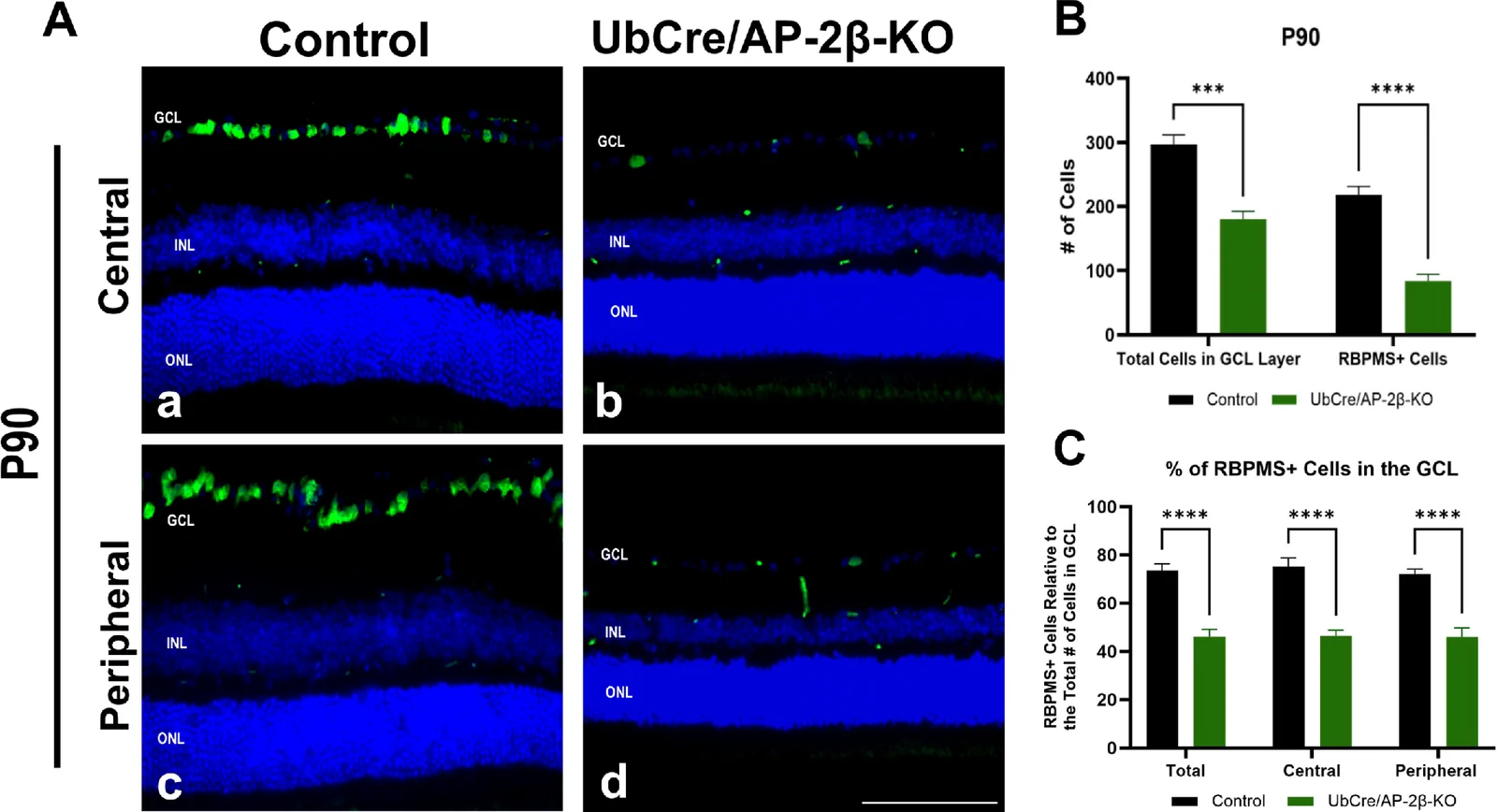
RBPMS and cell counts at P90. P22 to P25 UbCre/AP-2β-KO and control mice were injected with tamoxifen, and the eyes were enucleated at P90, processed, sectioned, and stained for RBPMS. (**A**) Immunofluorescence images of central and peripheral retinal sections from control and UbCre/AP-2β-KO mice at P90 stained for RBPMS (*green*) and nuclei (*blue*). (**B**, **C**) Quantification shows a reduction in RBPMS⁺ RGCs in UbCre/AP-2β-KO mice. Bar graphs display the total number of cells in the GCL layer (**B**) and the number and proportion of RBPMS⁺ cells (**C**) for P90. A two-way ANOVA was performed, and data were graphed using GraphPad Prism. *Error bars* represent SEM. ***P < 0.001, ****P < 0.0001 (*n* = 3). Magnification 40×. *Scale bar*: 100 µm.

At P90, both RBPMS⁺ RGC counts and total GCL cells were reduced in UbCre/AP-2β-KO mice (Fig. 11B). Control retinas contained 219 ± 13 RBPMS⁺ cells compared with 84 ± 11 cells in mutants (*P* < 0.001), indicating a significant loss of RGCs in the mutants (Fig. 11B). Total GCL cell counts were likewise significantly decreased in mutants, with controls showing 297 ± 15 cells compared to 180 ± 12 cells in UbCre/AP-2β-KO mice (*P* < 0.001) (Fig. 11B). Analysis of the proportion of RBPMS⁺ cells relative to total GCL cells further confirmed this reduction, as control mice exhibited 72.08% ± 2.13% in the peripheral retina, 75.20% ± 3.58% centrally, and 73.56% ± 2.81% overall, but mutants showed significantly reduced proportions of 46.04% ± 3.74%, 46.36% ± 2.49%, and 46.14% ± 3.09%, respectively (*P* < 0.01) (Fig. 11C). These findings show that, by P90, UbCre/AP-2β-KO mice exhibit a significant decline in both total GCL cell number and RBPMS⁺ RGCs, along with a reduction in the proportion of RBPMS⁺ cells across retinal regions (Fig. 11).

Given the reduction in RGCs, we also examined retinal layer thickness. At P60, UbCre/AP-2β-KO mice displayed a significant decrease in total retinal thickness compared with their control littermates with a mean decrease of 63.26 µm (*P* < 0.05), from 133.8 µm to 70.54 µm, further supporting the presence of glaucomatous changes (Fig. 12A). Analysis of subsequent individual retinal layers revealed that the outer nuclear layer (ONL) and inner nuclear layer (INL) significantly decreased in thickness in mutant eyes compared to controls (*P* < 0.05 and *P* < 0.01, respectively) (Fig. 12A). By P90, these deficits had further progressed, with KO mice exhibiting a greater mean decrease of 85.42 µm (*P* < 0.001) and persistent thinning of the ONL and INL in KO mice compared to controls (Fig. 12B). At this stage there was also a statistically significant decrease in the width of the inner plexiform layer (Fig. 12B).

**Figure 12.**
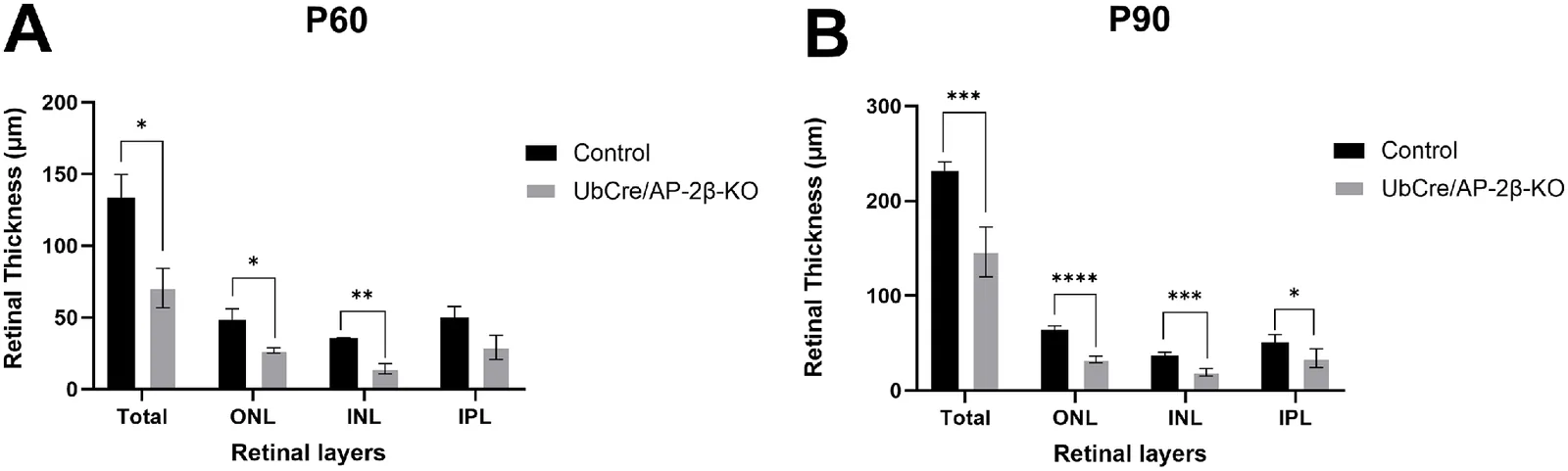
(**A**, **B**) The average retinal thickness (µm) of control and UbCre/AP-2β-KO mice at P60 (**A**) and P90 (**B**). P22 to P25 UbCre/AP-2β-KO and control mice were injected with tamoxifen, and the eyes were enucleated at P60 and P90, processed, sectioned, and stained with H&E. Retinal thickness was then measured. Retinal layers are displayed on the *x*-axis, with corresponding thickness measurements (µm) on the *y*-axis. An unpaired *t*-test was performed for each layer. *P < 0.05, **P < 0.01, ***P < 0.001, ****P < 0.0001. Figure was created in part using GraphPad Prism 9.0. Mean ± SD was calculated for all samples. P60 control, *n* = 3; P60 mutant, *n* = 3; P90 control, *n* = 4; P90 mutant, *n* = 4.

### Retinal Alterations in UbCre/AP-2β-KO Are Associated With Retinal Inflammation

RGC degeneration is accompanied by reactive Müller glial cells, astrocytes, and microglia, also known as gliosis, which can further amplify neuroinflammation and retinal degeneration.^35^ To determine the extent of neuroinflammation and its correlation with RGC cell loss in UbCre/AP-2β-KO, we evaluated glial reactivity in P90 control and mutant sections, a time point where a significant decrease in RGC cell numbers was observed in UbCre/AP-2β-KO retinas. Upregulation of glial fibrillary acidic protein (GFAP), an intermediate filament expressed in astrocytes and Müller glia that is usually overexpressed during retinal gliosis,^36^ was observed in both the central and peripheral retinas of UbCre/AP-2β-KO when compared to control retinas showing enhanced gliosis (Fig. 13). An increased expression of vimentin, another marker of Müller cells,^37^ and its colocalization with GFAP was observed in UbCre/AP-2β-KO retinas, thus indicating Müller glial activation in the mutant retinas.

**Figure 13.**
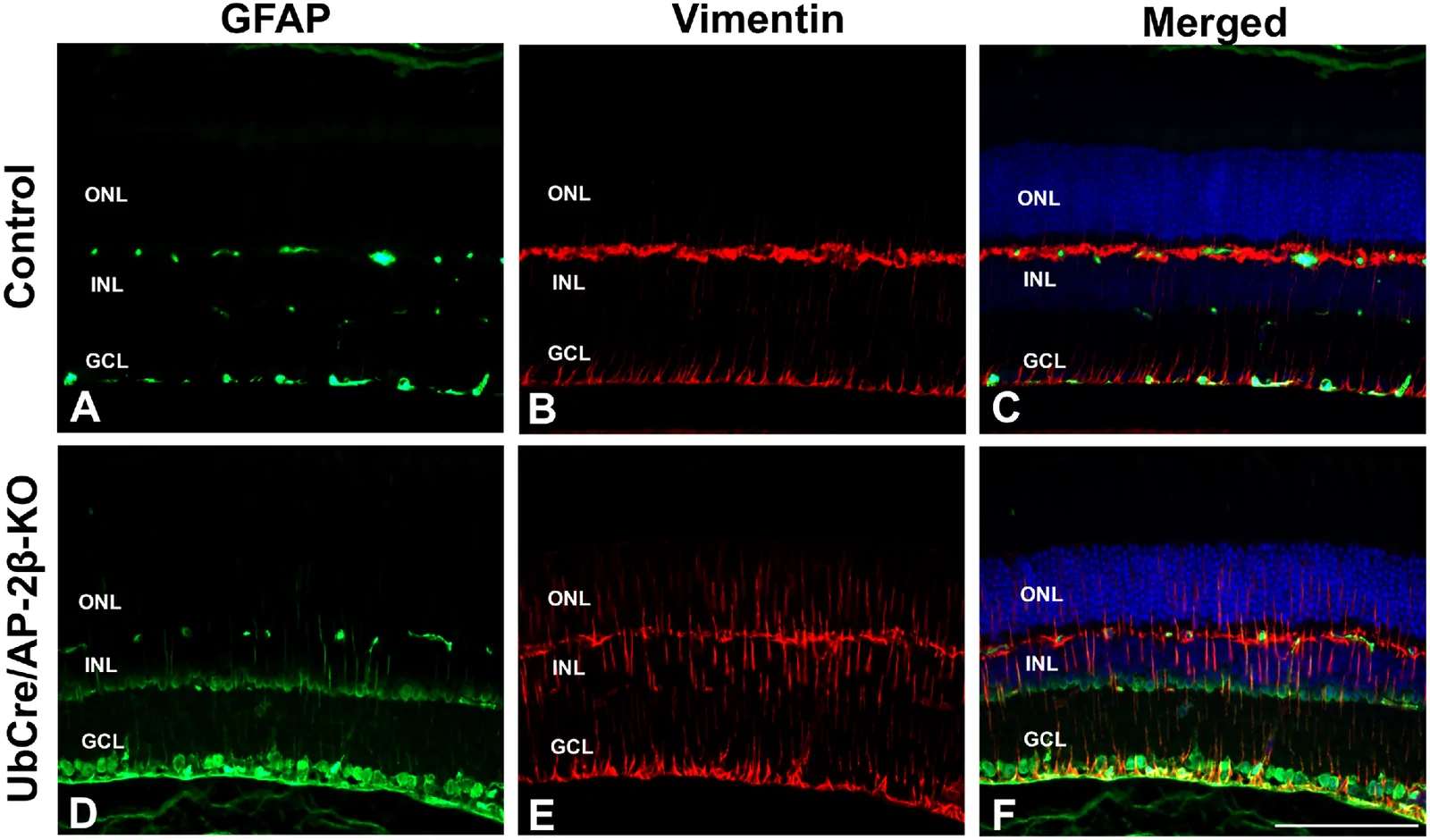
Glial reactivity in control and UbCre/AP-2β-KO retinas at P90. P22 to P25 UbCre/AP-2β-KO and control mice were injected with tamoxifen, and the eyes were enucleated at P90, processed, sectioned, and stained for GFAP and vimentin. (**A**–**F**) IHC staining of retinal sections from control and UbCre/AP-2β-KO mice at P90 showing GFAP (*green*) (**A**, **D**), vimentin (*red*) (**B**, **E**), and merged with DAPI (*blue*) (**C**, **F**). UbCre/AP-2β-KO retinas show increased GFAP signal extending into the INL, consistent with enhanced glial activation compared to controls. Magnification 40×. *Scale bar*: 200 µm.

## Discussion

Glaucoma is the leading cause of irreversible blindness globally, and understanding the genetic factors contributing to this disease is important in developing novel treatment approaches and improving patient outcomes. Our previous work has highlighted the importance of AP-2β as a key regulator for the development of the outflow-pathway structures, with its embryonic deletion disrupting TM and SC formation and leading to elevation of IOP and compromised retinal function.^22^^,^^23^ However, the role of AP-2β in IOP maintenance and disease onset in adult mice remained unknown. In the current study, we leveraged a tamoxifen-inducible ubiquitin–*Cre* recombinase system (*UbCre^ERT2^*) to induce conditional deletion of *Tfap2b* (UbCre/AP-2β-KO) at multiple, but defined, postnatal timepoints to delineate the temporal requirement of AP-2β in anterior angle development and adult IOP homeostasis.

Previously, our group created AP-2β NCC KO and AP-2β TMR KO mouse models in which the Wnt1 and MgpCre mouse lines were used to delete AP-2β from the cranial neural crest at E8.5 and POM cells at E14.5, respectively.^22^^,^^23^^,^^25^ The time points of AP-2β deletion in these mouse models were before the formation of TM anlage that starts between P1 and P328 when the POM cells placed at iridocorneal angle begin to differentiate into potential TM cells.^29^^,^^30^^,^^38^ Between P4 and P10, these cells progressively elongate and flatten as they begin to adopt the morphology of TM cells.^7^^,^^25^^,^^30^ The absence of the TM and SC and the presence of a partially closed angle phenotype in UbCre/AP-2β-KO mice, when compared to controls, treated with tamoxifen between P4 and P7 revealed that loss of AP-2β starting at P4 impairs the development of the anterior angle structures. The features observed in UbCre/AP-2β-KO were comparable to our previous model, AP-2β TMR KO,^23^ where early embryonic loss of AP-2β specifically in the POM led to an underdeveloped TM and SC, and angle closure.^23^ This phenotypic similarity between UbCre/AP-2β-KO and AP-2β TMR KO models reinforces that AP-2β is required during early postnatal development of the anterior angle structures. More specifically, our findings highlight an important role of AP-2β in the specification and differentiation of POM cells during early anterior angle development. Due to phenotypic similarity between P4 and P7 tamoxifen-treated UbCre/AP-2β-KO and our previously reported AP-2β TMR KO,^23^ we did not pursue additional evaluation of glaucomatous features including IOP elevation and RGC cell loss in P4 to P7 tamoxifen-treated UbCre/AP-2β-KO cohorts, as these effects were highlighted in the AP-2β TMR KO model.

Significant development and remodeling of the iridocorneal angle take place between P6 and P42, including flattening and elongation of the TM cells and further secretion of extracellular matrix fibers that are organized into lamellae or beams.^39^ Between P12 and P14, the TM matures, forming the characteristic beam-like architecture required for aqueous humor drainage.^29^^,^^30^^,^^38^ By P21, this remodeling produces definitive trabecular beams lined by thin, flattened endothelial cells.^38^ At the same developmental stage, the SC is also established, resulting in a functional conventional outflow pathway.^38^ Conditional deletion of AP-2β in UbCre/AP-2β-KO between P22 and P25 resulted in an anterior angle phenotype that appeared morphologically (H&E) similar to those of control mice, including an open iridocorneal angle as well as comparable expression of TM markers, in contrast to the phenotype observed following AP-2β deletion at P4 to P7 (Figs. 4 vs. 1). These findings indicate that the loss of AP-2β in adult mice does not disrupt the gross morphology of the TM. Despite the relatively normal appearance of the angle, UbCre/AP-2β-KO exhibited a significant increase in IOP relative to their control littermates. This demonstrated that AP-2β contributes to IOP homeostasis even in the context of an open-angle phenotype, possibly through modulation of iridocorneal angle structures including the TM and SC. Ultrastructural analyses of the iridocorneal angle region revealed irregular trabecular beams with increased inter-trabecular spaces, fragmented/decreased collagen fibers, and a more dispersed pigment in UbCre/AP-2β-KO compared to controls. These observations resemble previous findings reported for the iridocorneal angle region of *Cyp1b1* deficient mice.^40^ This gene is involved in metabolism of fatty acids, steroid hormones, and vitamins that has been implicated in primary congenital glaucoma.^40^ This genetic mouse model of glaucoma showed loss of collagen fiber associated with TM atrophy, and an irregular, reduced number of trabecular beams, irregular trabecular cells, and fragmented collagen fibers.^41^ The loss of collagen fibers is known to disrupt tissue elasticity and alter the cytoskeletal organization of TM cells, and this can compromise aqueous humor outflow and promote IOP elevation.^42^ The presence of irregular trabecular beams in UbCre/AP-2β-KO mutants compared with controls can be attributed to reduced and fragmented collagen fibers that may also be contributing to increased intertrabecular space (Fig. 9D vs. 9H). Moreover, a reduction in collagen fibers within the TM region of UbCre/AP-2β-KO also supports a role forAP-2β in modulating ECM protein expression. AP-2β has been recently reported to be specifically expressed in the TM3 population, a TM cell subtype that exhibits a beam-like morphology, between P6.5 and P14.^27^ The sparse population of TM cells in UbCre/AP-2β-KO suggests that AP-2β may be directly or indirectly involved in the maintenance of this specific TM population. In DBA/2J mice, a pigment dispersion glaucoma mouse model, an increased presence of pigment-laden macrophage-like cells accumulate within aqueous drainage channels and obstruct outflow leading to increased IOP.^43^ It is possible that the dispersed pigmented particles observed in the TM region of UbCre/AP-2β-KO represent a similar phenomenon and may contribute to elevated IOP.

In conventional outflow, the aqueous humor passes from juxtacanalicular TM across the inner wall of SC via transcellular and paracellular routes and is often associated with the formation of giant vacuoles in endothelial cells.^44^^,^^45^ The flattened giant vacuoles in the SC lumen of UbCre/AP-2β-KO may reflect increased stiffness of the inner wall endothelium, leading to impaired SC endothelial function and resulting in higher outflow resistance and thus ocular hypertension.^46^ Previous studies also found that mice with increased IOP possessed giant vacuoles that were notably smaller in size compared to controls, as well as increased thickness of the SC basement membrane and tighter adherens junctions, ultimately contributing to changes in aqueous humor outflow.^45^ Such changes are expected to elevate outflow resistance and ultimately contribute to ocular hypertension. Alternatively, it is possible that defects in the TM of this conventional pathway cause a significant reduction in fluid flow, preventing normal accumulation in the endothelial of SC. Recent studies have shown that there is relevant TM–SC crosstalk as the TM and ECM organization and stiffness regulate SC function through cell signaling.^47^^,^^48^ Regardless of the rationale, the cumulative structural abnormalities in the TM and SC we observed in the UbCre/AP-2β-KO eyes likely impaired aqueous outflow and contributed to the increased IOP. Together, our findings suggest that AP-2β expression is required for homeostasis in the TM region including TM cell, SC, and ECM components.

One notable finding was that the mutants within the same cohort displayed some variability in the magnitude of IOP elevation. Subsequent analyses revealed an inverse correlation between residual AP-2β expression and the degree of IOP elevation (Fig. 8), further supporting a dosage-dependent role for AP-2β in regulating IOP. The observed variability could be attributed to variable AP-2β deletion within the TM region, possibly in the TM3 subpopulation that is known to express AP-2β.^27^ In addition, this observation also highlights a limitation of this model with regard to efficiency of tamoxifen treatment–mediated recombination and excision of floxed alleles.^49^ Some of the UbCre/AP-2β-KO eyes that experienced increased IOP at P40 showed reduced IOP readings by P60 or P90. Interestingly, the eyes that failed to sustain elevated IOP displayed normal AP-2β expression at the later time point. This may be due to variability in recombination efficiency in distinct cells,^49^ with any mosaic inactivation allowing some TM cells to have limited AP-2β activity. These unrecombined TM cells could then repopulate the TM region and re-establish AP-2β expression. Indeed, this inference requires further evaluation. This variability may also explain the expression of AP-2β in other ocular tissues after tamoxifen exposure. In mice that were topically treated with tamoxifen, AP-2β expression was reduced in the TM region but appears to be expressed in other tissues, including the ciliary body and retina. This may be due to variability in recombination efficiency in different cell types, as well as the efficiency of the tamoxifen when applied as a topical eye drop. Further evaluation of the recombination efficiency may address these findings.

In both mouse and human, elevated IOP is known to exert pressure on the posterior segment, resulting in pressure-induced cell death of RGCs and thus impairing retinal function.^22^^,^^23^^,^^34^^,^^50^ RBPMS is a highly selective pan-RGC marker that labels essentially all RGC subtypes and can therefore be used to assess changes in the RGC population more comprehensively. Although the GCL predominantly contains retinal ganglion cells, it also includes a population of displaced amacrine cells.^51^ Consequently, in control mouse retinas, GFAP immunolabeling does not occur in 100% of the GCL nuclei because Brn3a selectively labels the ganglion cells but not displaced amacrine cells. The reduced proportion of RBPMS-expressing cells relative to the total GCL cell population in both central and peripheral retina at P60 in UbCre/AP-2β-KO mice suggests early signs of RGC degeneration or dysfunction, even though overall cell density remained unchanged. By P90, however, both RBPMS⁺ RGCs and the total number of GCL cells were significantly diminished in UbCre/AP-2β-KO mice, indicating a progression from early RGC alterations (i.e., at ∼P60) to overt cell loss.

These findings were further supported by Brn3a immunolabeling at P60 and P90 (Supplementary Figs. S7, S8). One of the established early markers of RGC degeneration in ocular hypertension models is the progressive loss of expression of Brn3a, a transcription factor and marker of RGC.^52^ Similar to the RBPMS findings, the reduced proportion of Brn3a-expressing cells relative to the total GCL cell population at P60 in UbCre/AP-2β-KO mice suggests early signs of RGC stress or dysfunction, even though overall cell density remained unchanged. (Supplementary Fig. S7). By P90, both Brn3a⁺ RGCs and the total number of GCL cells were significantly diminished (Supplementary Fig. S8). These findings are consistent with previous reports^53^^,^^54^ and further confirm that the loss of Brn3a expression precedes RGC cell death during glaucoma progression, such that early molecular changes are detected first and actual RGC loss becomes more evident at later stages. In addition, a significant reduction in retinal thickness in P60 and P90 UbCre AP-2β KO retinas when compared with control mice suggests that elevated IOP is leading to glaucomatous neurodegenerative defects in the retina. Our current findings are in conjunction with previous reports where increased IOP has been linked to retinal damage, including INL thinning.^55^^,^^56^

Glaucomatous neurodegeneration is driven not only by elevated IOP but also by factors such as ischemia, mitochondrial dysfunction, oxidative stress, and neuroinflammation that mainly involve glial cells, microglia, astrocytes, and Müller cells.^57^ The increase in IOP is known to activate gliosis in both astrocytes and Müller cells and is marked by overexpression of structural proteins GFAP^58^ and vimentin.^59^ Chronic activation of glial cells leads to a proinflammatory retinal environment that disrupts the blood–retinal barrier and contributes to RGC death.^57^ The increased expression of GFAP and vimentin and their colocalization across the retinal layers in 3-month-old UbCre AP-2β KO mice indicate increased glial reactivity and retinal stress. Furthermore, the observed gliosis in P90 UbCre AP-2β KO mice also shows that both astrocytes and Müller cells remain functionally active. In contrast to our previous AP-2β NCC KO closed-angle glaucoma model, the more moderate IOP changes in the UbCre/AP-2β-KO mice suggest a progressive increase in glial response over time, better mirroring the progressive nature of an open-angle glaucoma phenotype.^35^ The extent of retinal degeneration observed in this model appears to correlate with the degree of AP-2β loss within the TM region. Greater deletion was associated with higher IOP that consequently induced retinal defects, including RGC loss, retinal thickness, and inflammation (data not shown).

Although our goal was to restrict AP-2β knockout to the anterior segment, the use of IP injections or topical drops of tamoxifen raises the possibility of off-target effects. In addition, we believe that topical tamoxifen administration is unlikely to affect the retina to the same extent as systemic tamoxifen exposure in UbCre/AP-2β-KO, given the limited ocular penetration associated with topical delivery. AP-2β is expressed in multiple ocular tissues, including the cornea, lens, ciliary body, and retina.^15^^,^^19^ Therefore, it could be speculated that the observed retinal defect may be due to the loss of AP-2β in the retina. However, our previous work has shown that simultaneous loss of AP-2α and AP-2β is required for complete loss of horizontal cells, disrupted amacrine cell mosaics, defective photoreceptor synapses, and outer plexiform layer thinning, ultimately impairing retinal function.^60^ In contrast, individual deletion of either AP-2α or AP-2β in the retina produced no effect, as amacrine and horizontal cell populations were maintained.^16^^,^^20^^,^^60^ Based on these and our current findings, we suggest that the deletion of AP-2β alone is unlikely to cause RGC loss, further demonstrating that the retinal changes observed in our model are primarily driven by increased IOP rather than direct effects on the RGCs.

Several mouse models of open-angle glaucoma have been established to investigate glaucoma progression, including mechanically induced models such as the optic nerve crush and microbead models, as well as models that develop spontaneous mutations resulting in glaucomatous features such as the DBA/2J model.^61^ Furthermore, targeted gene deletion models also provide critical insights into glaucoma progression. Mice harboring mutations in *Sh3pxd2b*,^62^ *Pitx2*,^63^ *Foxc1*, *Foxc2*,^12^ and *Lmx1b*,^64^ as well as the AP-2β NCC KO and AP-2β TMR KO strains generated by our group,^23^^,^^22^ all display anterior segment dysgenesis, highlighting the role of these genes in the etiology and pathophysiology of a particular subtype of glaucoma. In contrast, here we have demonstrated for the first time, to our knowledge, that temporal deletion of *Tfap2b* using tamoxifen-inducible *Ubc-*Cre.KI mice produces two distinct glaucoma phenotypes, depending on the developmental stage at which *Tfap2b* is deleted. Loss of AP-2β beginning at P4, prior to the maturation of anterior outflow structures, resulted in a more severe phenotype including a peripheral anterior synechiae–like angle closure driven by defective anterior segment development and absence of both the TM and SC, resembling AP-2β TMR KO.^23^ Conversely, the deletion of AP-2β starting at P22 (i.e., after functional anterior angle structures are formed) resulted in an open iridocorneal angle, with both the TM and SC intact. Despite this open-angle phenotype, the loss of AP-2β at this time point resulted in increased IOP and progressive retinal degeneration and inflammation, indicating that UbCre/AP-2β-KO mice could serve as a useful model of human POAG. Given the association of the human *TFAP2B* gene with POAG,^28^ the UbCre/AP-2β-KO mouse model can serve as an excellent system to explore mechanisms through which AP-2β regulates TM function and thus IOP homeostasis. As a potential model of POAG, UbCre/AP-2β-KO mouse can also be employed to examine the responses and underlying mechanisms of IOP-lowering therapies.

## Supplementary Material

Supplement 1
