# Supplementary material for: Temporal Deletion of AP-2β in Postnatal Mice Demonstrates Distinct Roles in Anterior Angle Development and Intraocular Pressure Homeostasis
Source: Invest Ophthalmol Vis Sci. 2026 Oct 28;67(12):70. doi: 10.1167/iovs.67.12.70 (PMC13722983; doi:10.1167/iovs.67.12.70)
Supplement: Supplementary file 1 [file iovs-67-12-70_s001.doc]

**Supplementary Index:**

Supplementary Figure S1. Cornea thickness in UbCre/AP-2β-KO and controls. (A) Comparison in cornea thickness of P14 mutant and control mice treated with tamoxifen from P4-P7. (B) Comparison of cornea thickness in P90 mutant and control mice treated with tamoxifen from P22-P25. Error bars represent the standard error of the mean (SEM). n=3 for each group. Ns, no significant change between genotypes.

Supplementary Figure S2. IHC staining for myocilin (red) and α-SMA (green) in P14 UbCre/AP-2β-KO and control mice. P4-P7 UbCre/AP-2β-KO and control mice were topically treated with tamoxifen treatment and the eyes were enucleated, fixed, sectioned and stained. The asterisks show the absence of the TM in the mutant mice compared to controls. n=3. TM, trabecular meshwork; CB, ciliary body; CM, ciliary muscle. Scale bars 100 µm (40X).

Supplementary Figure S3. IHC staining for endomucin (red) in P14 UbCre/AP-2β-KO and control mice. P4-P7 UbCre/AP-2β-KO and control mice were topically treated with tamoxifen treatment and the eyes were enucleated, fixed, sectioned and stained. Staining shows the absence of the SC in mutant mice compared to controls. n=3. TM, trabecular meshwork; CB, ciliary body; C, cornea; R, retina. Asterisk (*) marks SC.  Scale bars 100 µm (40X).

Supplementary Figure S4. IHC staining for myocilin (red) and α-SMA (green) in P40 UbCre/AP-2β-KO and control mice. P22-P25 UbCre/AP-2β-KO and control mice were treated with tamoxifen treatment and the eyes were enucleated, fixed, sectioned and stained. Staining shows the presence of the TM in both the control and mutant mice. n=3. TM, trabecular meshwork; CB, ciliary body; CM, ciliary muscle. Scale bars 100 µm (40X).

Supplementary Figure S5. IHC staining for endomucin (red) in P40 UbCre/AP-2β-KO and control mice. P22-P25 UbCre/AP-2β-KO and control mice were treated with tamoxifen treatment and the eyes were enucleated, fixed, sectioned and stained. Staining shows the presence of the SC in both the control and mutant mice. n=3. TM, trabecular meshwork; CB, ciliary body; C, cornea; R, retina. Asterisk (*) marks SC.  Scale bars 100 µm (40X).

Supplementary Figure S6. AP-2β expression quantification in the anterior angle of control and UbCre/AP-2β-KO mice at P60. P22-P25 UbCre/AP-2β-KO and control mice were injected with tamoxifen, eyes were measured for IOP, and the eyes were enucleated at P60, processed, sectioned and stained with AP-2β, and quantified through FIJI. A two-way Anova was performed for genotype. Statistical significance is indicated using standard asterisk notation (*p<0.05, *** p<0.001). Figure created in part using GraphPad Prism 9.0. Mean ± standard error of the mean (SEM) was calculated for all samples. (Control: n=3; UbCre/AP-2β-KO Low IOP n=5; UbCre/AP-2β-KO High IOP: n = 4).

Supplementary Figure S7. Brn3a staining and RGC cell counts at P60. P22-P25 UbCre/AP-2β-KO and control mice were injected with tamoxifen and the eyes were enucleated at P60, processed, sectioned and stained for Brn3a. (A) Immunofluorescence images of central and peripheral retinal sections from control and UbCre/AP-2β-KO mice at P60 stained for Brn3a (red) and nuclei (blue). Quantification shows a reduction in Brn3a⁺ retinal ganglion cells in UbCre/AP-2β-KO mice. Bar graphs display the total number of cells in the GCL layer (B) and the number and proportion of Brn3a⁺ cells (C) for P60. Two-way Anova was performed, and data was graphed using GraphPad Prism. Error bars represent standard error of the mean (SEM). Significance levels: p<0.01 (**), p<0.0001 (****). n=3. Scale bar: 100 µm (40x). GCL, ganglion cell layer; INL, inner nuclear layer; ONL, outer nuclear layer.

Supplementary Figure S8. Brn3a staining and RGC cell counts at P90. P22-P25 UbCre/AP-2β-KO and control mice were injected with tamoxifen and the eyes were enucleated at P90, processed, sectioned and stained for Brn3a. (A) Immunofluorescence images of central and peripheral retinal sections from control and UbCre/AP-2β-KO mice at P90 stained for Brn3a (red) and nuclei (blue). Quantification shows a reduction in Brn3a⁺ retinal ganglion cells in UbCre/AP-2β-KO mice. Bar graphs display the total number of cells in the GCL layer (B) and the number and proportion of Brn3a⁺ cells (C) for P90. A two-way anova was performed, and data was graphed using GraphPad Prism. Error bars represent standard error of the mean (SEM). Significance levels: p<0.01 (**), p<0.001 (***). n=3. Scale bar: 100 µm (40x). GCL, ganglion cell layer; INL, inner nuclear layer; ONL, outer nuclear layer.
